# Case Report: Transrectal ultrasound in adolescent Müllerian anomalies - bridging the gap between conflicting international guidance and real-world clinical pragmatism

**DOI:** 10.3389/frph.2026.1791559

**Published:** 2026-04-14

**Authors:** Szabolcs Bozsa, Rasiah Bharathan, Maria Monsorno-Dechant, Theresa Steindl-Tomschizek, Christian Neumann, Istvan A. Harmati, Zoltan Nemeth

**Affiliations:** 1 Department of Obstetrics and Gynecology, Faculty of Health and Sport Sciences, Széchenyi István University, Győr, Hungary; 2 Department of Gynecology, Hospital of the Brothers of St. John of God, Vienna, Austria; 3 Division of General Gynecology and Gynecological Oncology, Department of Obstetrics and Gynecology, Comprehensive Cancer Center Vienna, Medical University of Vienna, Vienna, Austria; 4 Department of Radiology and Nuclear Medicine, Hospital of the Brothers of St. John of God, Vienna, Austria; 5 Department of Systems Science, Széchenyi István University, Győr, Hungary

**Keywords:** adolescent, clinical decision-making, endosonography, health services accessibility, Müllerian ducts, practice guideline as topic, unicornuate uterus

## Abstract

An 18-year-old patient with an intact hymen presented with severe, cyclical dysmenorrhea, representing a common diagnostic challenge. Initial evaluation at an outside institution, including transabdominal ultrasound (TAUS) and magnetic resonance imaging (MRI), yielded an inaccurate initial radiological report, creating a diagnostic impasse. As transvaginal ultrasound (TVUS) is not feasible in this patient group, point-of-care transrectal ultrasound (TRUS) was performed, leveraging its superior near-field resolution, providing the definitive diagnosis of a non-communicating rudimentary uterine horn. The diagnosis established by TRUS was corroborated by intraoperative findings at laparoscopy. Surgical resection of the rudimentary horn resulted in complete and sustained symptom resolution, validating the clinical decision to pursue further investigation. This case, involving a routine application of a well-established technique, illustrates the diagnostic gap created by conflicting international guidance. This disconnect between recommendations and necessary clinical practice is particularly challenging in low-resource settings where advanced imaging is inaccessible. To address this uncertainty, we propose a pragmatic diagnostic algorithm positioning TRUS as a second-line, problem-solving modality. The pathway applies when initial non-invasive evaluation with TAUS and transperineal ultrasound (TPUS) is inconclusive due to limited fundal visualization. This highlights a key paradox: while guidance frameworks champion patient-centered principles, their rigid interpretation can create practical barriers in outlier cases. TRUS is then offered as a potential diagnostic step for consenting older adolescents when access to expert-interpreted MRI is either delayed or unavailable. This framework provides a clear pathway for formal studies to generate the evidence currently lacking in this specific population.

## Introduction

Congenital Müllerian anomalies are rare developmental disorders, often undiagnosed due to asymptomatic presentation or diagnostic limitations (1). Among these, the unicornuate uterus is a rare anomaly, though its prevalence increases in subfertile populations (2, 3). A non-communicating rudimentary horn with functional endometrium is present in 20%–25% of unicornuate uterus cases (4). However, the true prevalence and clinical spectrum are likely under-recognized due to their rarity, limited clinical exposure, and the coexistence of multiple classification systems (1).

To harmonize diagnostic terminology, several classification systems have been introduced. The European Society of Human Reproduction and Embryology (ESHRE)/European Society for Gynaecological Endoscopy (ESGE) system proposed a new anatomical framework, renaming the unicornuate uterus as ‘hemiuterus' (Class U4) (5). In contrast, the American Society for Reproductive Medicine (ASRM) MAC2021 classification expanded the traditional framework, retaining the term ‘unicornuate uterus’ while describing five morphological variants (1). These differing approaches reflect persistent challenges in clinical taxonomy and interobserver agreement (6), underscoring the absolute necessity of high-resolution imaging to precisely delineate the uterine cavity and fundal contour for accurate categorization.

Accurate preoperative diagnosis is essential for surgical management and preventing severe long-term complications like hematometra, endometriosis, and impaired fertility (3, 4, 7). The standard imaging pathway in pediatric and adolescent gynecology typically begins with ultrasound, leveraging its non-invasive nature and accessibility (8). According to the 2015 Thessaloniki ESHRE/ESGE consensus, initial evaluation should include a gynecological examination and two-dimensional (2D) ultrasound for asymptomatic patients, with three-dimensional (3D) ultrasound or magnetic resonance imaging (MRI) recommended for symptomatic or complex cases (9). MRI is considered the most accurate non-invasive modality, while 3D- ultrasound shows high sensitivity in experienced hands (10, 11).

A significant diagnostic challenge arises in adolescent patients with an intact hymen, where transvaginal ultrasound (TVUS) is typically contraindicated. The two leading international professional bodies offer conflicting and ultimately divergent guidance for this specific scenario. On one hand, the Thessaloniki ESHRE/ESGE consensus acknowledges transrectal ultrasound (TRUS) as a technically feasible alternative for evaluating the uterus in cases of vaginal obstruction, but then explicitly excludes its use in adolescents (“not in children nor in adolescents”), without providing a specific rationale for this restriction (9). On the other hand, the 2025 guidance from the British Medical Ultrasound Society (BMUS), referencing the National Institute for Health and Care Excellence (NICE), goes further and categorically rejects TRUS as a suitable alternative to TVUS, stating, “There is no evidence to indicate that transrectal ultrasound is a reasonable or acceptable alternative to TVUS” (12).

This direct conflict—where one body implicitly permits the technique but excludes the patient group, while the other rejects the technique entirely—creates a clear diagnostic gap. Consequently, clinicians are left in a practical void: when initial non-invasive evaluation with transabdominal (TAUS) and transperineal (TPUS) ultrasound is inconclusive, they lack a recommended point-of-care ultrasound pathway, as the next suggested step—timely, expert-interpreted MRI—is often limited by access and availability, a barrier that disproportionately affects patients in resource-limited environments.

We report the successful use of TRUS in an 18-year-old symptomatic patient with an intact hymen with a non-communicating functional rudimentary uterine horn, where standard imaging pathways were inconclusive. Using this case as a framework, we challenge the rationale behind vague, restrictive recommendations and advocate for a pragmatic, point-of-care, and patient-centered diagnostic approach when TVUS is contraindicated and first-line imaging fails.

## Case description

An 18-year-old nulliparous patient with an intact hymen [Body mass index (BMI): 31.6 kg/m^2^] presented with severe cyclical dysmenorrhea of one year [Visual Analogue Scale (VAS) 10/10], impairing her quality of life. Menarche occurred at age 11, followed by a multi-year period of secondary amenorrhea concurrent with a now-resolved episode of anorexia nervosa. Menstrual cycles resumed at 17, regular since. Analgesics provided minimal relief, and hormonal therapy had not been trialed. At our tertiary center, hormonal treatment with dienogest was proposed but declined in favor of definitive surgical management. The timeline of the patient's clinical course is summarized in Table 1.

**Table 1:** Timeline of the episode of care, detailing the diagnostic trajectory and surgical management.

Timepoint	Clinical Event and Intervention
Age 11	Menarche.
Age 11–17	Secondary amenorrhea (attributed to anorexia nervosa). No pelvic symptoms.
Age 17	Resumption of regular menstrual cycles.
Age 17–18	Onset of severe cyclical dysmenorrhea (VAS 10/10), unresponsive to analgesics.
Initial Work-up *(Outside Institution)*	Transabdominal ultrasound and MRI performed. Results inconclusive (misinterpreted as necrotic fibroids or ovarian tumor).
Day 0 *(Tertiary Center)*	Patient presentation. TVUS contraindicated (intact hymen). Point-of-care Transrectal Ultrasound (TRUS) performed. Diagnosis: Hemiuterus with non-communicating rudimentary horn and hematometra.
Preoperative Period	Retrospective expert review of previous MRI confirmed the TRUS findings. Surgery scheduled.
Surgery	Laparoscopic resection of the rudimentary horn and right salpingectomy.
Post-op Week 10	Complete resolution of dysmenorrhea. PGI-I score: 1 (“very much better”).
Post-op Year 1	Sustained symptom resolution. No analgesic use required.

MRI, magnetic resonance imaging; PGI-I, Patient Global Impression of Improvement; TAUS, transabdominal ultrasound; TRUS, transrectal ultrasound; TVUS, transvaginal ultrasound; VAS, visual analogue scale.

### Diagnostic assessment

Initial TAUS and a subsequent MRI performed at an outside institution suggested necrotic fibroids or an ovarian tumor. The patient was referred to our tertiary referral gynecological center, the Hospital of the Brothers of St. John of God in Vienna, Austria. At the time of her presentation to our tertiary center, the previous imaging studies were not available for immediate review, creating a diagnostic impasse. A repeat gynecological examination was therefore performed. As the hymen was intact, TVUS was not feasible. With informed consent, TRUS was performed under strict safeguarding protocols, including a female clinical chaperone and a trauma-informed approach allowing the patient to halt the procedure at any time. The examination utilized a Voluson S6 ultrasound system (GE Healthcare, Zipf, Austria) equipped with a 2D endocavity probe (IC9-RS; 4.0–9.0 MHz). Following the standard protocol for endosonography to ensure optimal visualization, the examination was performed with an empty bladder. Notably, no specific bowel preparation was required or administered, which further underscores the feasibility of this modality in an acute, point-of-care setting. The probe was lubricated and gently introduced into the rectum, and the scanning technique mirrored that of TVUS.

**Figure 1:**
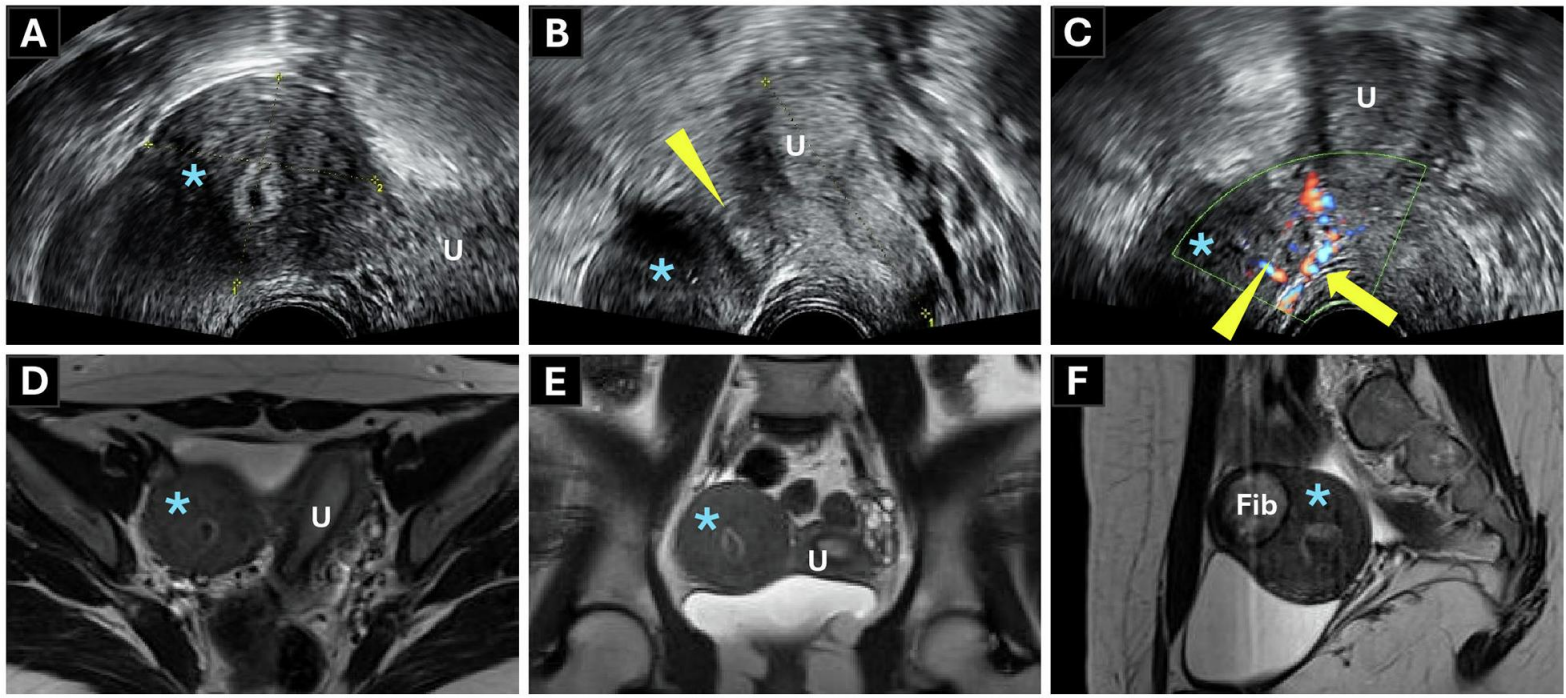
Preoperative multimodal imaging. **(A–C)** transrectal ultrasound (TRUS) findings. **(A)** Transverse view delineating the left-sided hemiuterus (U) and the adjacent rudimentary horn with hematometra (star). **(B)** The fibromuscular band (arrowhead) connecting the two structures. **(C)** Color Doppler imaging demonstrating a separate arterial branch (arrowhead) from the main uterine vessels (arrow) supplying the horn. **(D–F)** T2-weighted magnetic resonance imaging (MRI) scans confirming the TRUS findings. **(D)** Transverse view showing the hemiuterus (U) and rudimentary horn (star). **(E)** Coronal view confirming the absence of communication. **(F)** Sagittal view demonstrating the associated fibroid (Fib).

TRUS showed an anteverted, tubular uterus (67 × 33 × 33 mm) deviated to the left, consistent with a hemiuterus with homogenous 6 mm endometrium. Adjacent to the uterus, a 41 × 44 mm non-communicating rudimentary horn was seen with hematometra, and a 37 × 33 mm ventral fibroid. No communication was evident between the horn and the uterine cavity. Color Doppler imaging demonstrated the right uterine artery supplying the hemiuterus, with a branch to the rudimentary horn (Figures 1A–C). Avascular fibromuscular tissue (21 mm) connected the two structures. The left ovary appeared normal O-RADS 1 (Ovarian-Adnexal Reporting and Data System classification), while the right ovary was not visualized. Because the intact hymen precluded a physical vaginal examination, the presence of a single external cervical os was identified exclusively via TRUS. Additionally, a concurrent renal ultrasound demonstrated normal urinary tract anatomy, ruling out associated renal anomalies.

Retrospective MRI review by a gynecological radiology consultant confirmed the TRUS findings: left hemiuterus, right rudimentary horn with hematometra, and no evidence of communication (Figures 1D–F) or urinary tract anomalies. The diagnosis was classified as Class U4a C0 V0 per ESHRE/ESGE criteria (5) and as a unicornuate uterus with functional non-communicating horn per MAC2021 (1).

### Therapeutic intervention and outcomes

**Figure 2:**
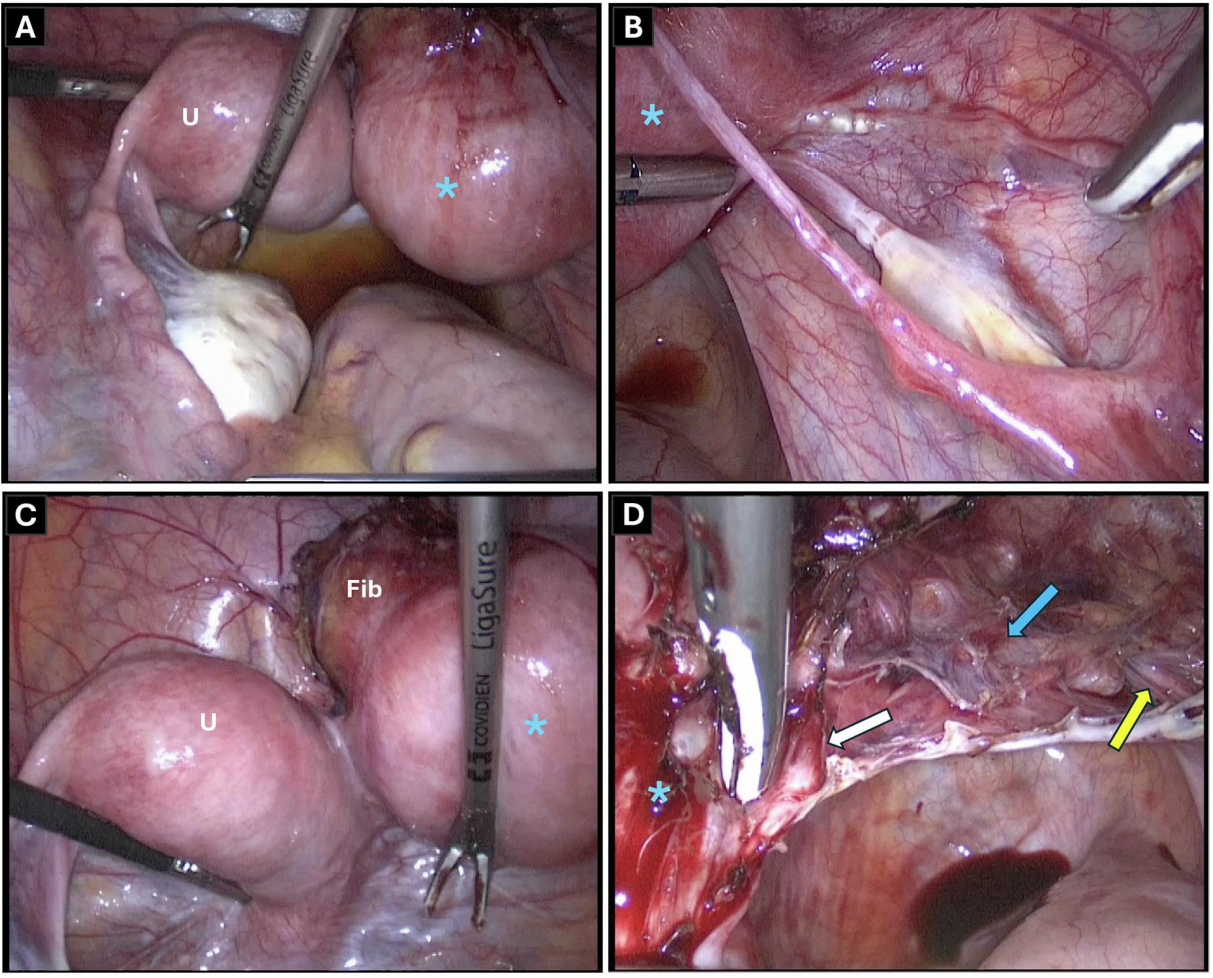
Operative findings. Laparoscopic views of the pelvic anatomy. **(A)** Normal left adnexa. **(B)** Hypoplastic right adnexa. **(C)** The unicornuate uterus (U), the non-communicating rudimentary horn (star) with the ventral fibroid (Fib), and the connecting fibromuscular band. **(D)** Retroperitoneal dissection showing the right uterine artery (blue arrow) crossing the ureter (yellow arrow) and giving off the branch to the rudimentary horn (white arrow).

Laparoscopy confirmed the anatomical findings, showing a hypoplastic right adnexa, a normal left adnexa and fibroid in the rudimentary horn (Figures 2A–C). A clear dissection plane was identified between the rudimentary right horn and the unicornuate hemiuterus. No endometriosis was present. Resection was performed using LigaSure (Medtronic, Minneapolis, MN, USA) to ensure optimal hemostasis while minimizing thermal spread to the adjacent functional hemiuterus and its vascular supply. Following right salpingectomy and ureter mobilization, the arterial branch to the horn was divided while preserving perfusion to the hemiuterus (Figure 2D). The fibromuscular tissue was transected, and the rudimentary horn was resected. The uterine serosa was closed with interrupted 2-0 monofilament absorbable sutures. The 80 g specimen was extracted via an endobag. The postoperative course was uneventful, with no complications or readmission.

Histopathology confirmed functional endometrium within myometrial tissue, consistent with a non-communicating horn. At 10-week follow-up, the patient reported complete symptom resolution, regular menstruation, and a Patient Global Impression of Improvement (PGI-I) score of 1 (“very much better”). At one-year postoperative telephone follow-up, the patient remained completely asymptomatic, with regular menstruation, no recurrence of pelvic pain, and no need for analgesic medication. Postoperatively, the patient received targeted reproductive counseling regarding the long-term obstetric risks of a hemiuterus, specifically preterm birth and fetal malpresentation.

## Discussion

This case illustrates a common diagnostic dilemma: evaluating pelvic pathology in an adolescent with an intact hymen where standard imaging pathways are inconclusive. Our experience supports repositioning TRUS not as a routine substitute for TVUS, but as a potential, problem-solving modality in a well-defined clinical niche.

### Strengths and limitations

The primary strength of this approach lies in the utilization of a readily available, point-of-care modality (TRUS) to resolve a diagnostic impasse created by inconclusive non-invasive imaging. This allowed for immediate diagnosis and surgical planning at the point of care, avoiding the logistical delays associated with retrieving external imaging for specialist re-evaluation. Furthermore, real-time TRUS provided immediate, dynamic information regarding organ mobility and tissue characteristics that static MRI interpretation alone cannot offer, thereby facilitating prompt decision-making in a symptomatic patient. A limitation of this report is that it represents a single case performed by highly specialized operators, meaning the results may not be universally reproducible without similar expertise; however, the diagnostic challenge presented is prototypic of the adolescent population. Additionally, while the initial MRI was inconclusive, the retrospective expert review confirmed the TRUS findings, highlighting the operator-dependency of both modalities.

### The diagnostic gap: when standard imaging fails

Accurate diagnosis of congenital uterine anomalies is paramount for guiding prognosis and management (13). While TVUS is the first-line modality, its contraindication in adolescents with an intact hymen creates a diagnostic gap. In our case, both TAUS and initial MRI were misinterpreted, leading to diagnostic delay. This is not uncommon. While TAUS is often limited by patient habitus (8), this limitation was highly relevant in our case, as the patient's BMI of 31.6 kg/m^2^ likely compromised acoustic windows and contributed to the initial diagnostic ambiguity. Furthermore, the diagnostic accuracy of MRI, frequently cited as the gold standard, can itself be highly conditional.

This highlights that the diagnostic power of MRI lies not just in the technology but also in multidisciplinary expertise that is not universally available. Leading professional documents from both the radiological and clinical communities underscore this point. Specifically, the European Society of Urogenital Radiology (ESUR) and the ESHRE/ESGE consensus both stipulate that for optimal results, MRI interpretation should be a collaborative process between an imaging expert and an experienced gynecologist (9, 14). Our case, where an initial MRI interpretation was inconclusive, exemplifies this real-world scenario (11). This reality exposes a critical gap: the ‘gold standard’ is contingent not only on the availability of MRI technology itself but also on a multidisciplinary expertise that is not universally accessible. As our case demonstrates, even in high-resource healthcare systems, the lack of immediate, expert-level radiological interpretation can create significant diagnostic delays. This diagnostic bottleneck can be significantly exacerbated in low-resource settings, where the scarcity of both advanced imaging hardware and expert interpretation may render MRI-dependent algorithms highly impractical. It is precisely this gap where the value of ultrasound performed by the treating clinician, who can integrate clinical findings in real-time, becomes paramount. Furthermore, real-time ultrasound offers the distinct advantage of dynamic assessment—such as evaluating organ mobility (‘sliding sign’) or site-specific tenderness—providing functional data that complements static cross-sectional imaging.

### TRUS: a point-of-care solution for a diagnostic dilemma

TRUS overcomes many limitations of initial non-invasive approaches (TAUS/TPUS) by placing a high-frequency transducer in close proximity to the pelvic organs, providing anatomical detail comparable to TVUS. In our patient, TRUS was the pivotal step that correctly identified the hemiuterus, the non-communicating horn, and its vascular supply, enabling accurate preoperative planning. This aligns with existing literature demonstrating the high diagnostic yield of TRUS in select cases. Its use as a valid diagnostic tool in large, modern cohorts of patients with Müllerian anomalies further normalizes its application in clinical practice (3, 15). This approach is validated by studies that faced a similar diagnostic gap in patients in whom transvaginal access was not feasible. In a large cohort where TVUS was unacceptable and initial non-invasive ultrasound proved insufficient, it was demonstrated that TRUS is diagnostically equivalent to TVUS for high-resolution morphological assessment (16). Crucially, the same literature highlights the established role of TRUS in diagnosing Müllerian anomalies, directly linking the validated technique to our clinical scenario. Furthermore, its use has been validated for diagnosing complex Müllerian anomalies in the same patient population (17).

Furthermore, when compared to other second-line alternatives, TRUS offers distinct advantages. While guidance documents also suggest TPUS, this often implies the use of 3D technology for optimal uterine assessment (9), which carries its own limitations regarding cost and expertise. In contrast, TRUS can provide excellent anatomical detail of the uterus and cervix even with a standard 2D endocavity probe, as demonstrated in our case. While 3D ultrasound, including 3D TRUS, is considered optimal for obtaining the coronal plane for definitive classification (15), our case demonstrates that a conclusive diagnosis is often achievable with ubiquitous 2D technology alone. While transperineal imaging is valuable for the distal genital tract, its resolution for detailed fundal assessment can be limited by the depth of the field and acoustic shadowing. In this specific context, TRUS offers superior visualization of the uterine corpus, making it an anatomically more logical choice when a uterine anomaly is suspected.

### Deconstructing the guidance paradox: balancing evidence and individualized care

It is crucial to acknowledge the valid and legitimate concerns that likely underpin the restrictive stance of current guidance documents regarding TRUS in adolescents. Professional bodies must prioritize population-level safety, and the reluctance to endorse TRUS is undoubtedly rooted in the need to protect a vulnerable demographic from potential psychological distress, physical discomfort, and the risk of coercion. Furthermore, TRUS is highly operator-dependent; without specific expertise, there is a significant risk of misdiagnosis, which could lead to inappropriate surgical planning. These are profound ethical and clinical considerations. However, as this case illustrates, in a capable, consenting patient, these valid concerns can be effectively mitigated through rigorous pre-procedural counseling, a trauma-informed approach allowing the patient to halt the procedure at any time, the presence of a clinical chaperone, and execution by an experienced operator. The tension lies in applying population-protective guidelines to an individual clinical impasse.

A critical nuance in this case—and a major flaw in current recommendations—is the complex semantic and legal entanglement surrounding the term ‘adolescent’. According to the contemporary, internationally recognized framework (18), adolescence spans from 10 to 24 years. If we strictly apply the ESHRE/ESGE restriction (“not in children nor in adolescents”) (9), our 18-year-old patient—and indeed any patient up to age 24—is excluded from TRUS based purely on a developmental classification. This creates a profound ethical paradox: it uses a broad biological term to strip a legally adult woman of her autonomy to consent to a clinically indicated medical procedure. The ESHRE/ESGE consensus, by excluding this demographic without explicit medical justification (9), relies on a strict, rule-based model that imposes a blanket restriction. In contrast, the BMUS guidance embodies a modern, patient-autonomy-based model (12), explicitly stating that individuals aged 16 and over (and mature minors under 16 with Gillick competence) have the legal capacity to consent to intimate examinations.

However, BMUS creates its own diagnostic void by categorically rejecting TRUS as a modality. To resolve this diagnostic and ethical conundrum, we argue that the threshold for performing TRUS should not be dictated by the vague biological umbrella term of ‘adolescence’. Instead, it must be governed by two concrete pillars: (1) strict clinical indication and (2) the patient's legal capacity to provide informed consent. Therefore, our proposed pathway is explicitly targeted at older adolescents (late adolescence, 15–19 years) and young adults who possess this legal capacity—such as our 18-year-old legal adult—provided rigorous safeguarding protocols are maintained. For younger adolescents (e.g., <16 years without established Gillick competence) lacking this legal and psychological capacity, we strongly advocate that MRI remains the mandatory, non-negotiable standard.

While adherence to evidence-based guidance is the cornerstone of standard practice, the rigid application of population-based recommendations in complex, outlier cases can paradoxically compromise patient care by causing diagnostic delays. Given that current guidance states every person… aged 16 years or older is eligible to be offered TVUS” (12), it presents a logical inconsistency to endorse intimate examinations for consenting adults while categorically rejecting an anatomically appropriate alternative (TRUS) when vaginal access is precluded. Patient tolerability is high with appropriate counseling, with studies suggesting TRUS is well-tolerated and less distressing than anticipated (19), and anatomical risks are also minimal, with the endocavity probe's diameter comparable to standard rectoscopic instruments, for which extensive experience suggests an extremely low risk of rectal injury (20). Ultimately, this aligns with the principle that “the concept of virginity plays no part in the clinical decision making,” a tenet that should logically extend to all anatomically appropriate imaging modalities when clinically indicated (12).

This highlights a broader, well-recognized challenge in guideline development: how to reconcile a rigorous evidence-based approach with the pragmatic needs of clinicians facing diagnostic uncertainty in specific, uncommon scenarios (6). Our case suggests that in such situations, a clinician's judgment and the use of anatomically logical tools like TRUS may play a crucial role.

### Navigating conflicting guidance: a pragmatic proposal

**Figure 3:**
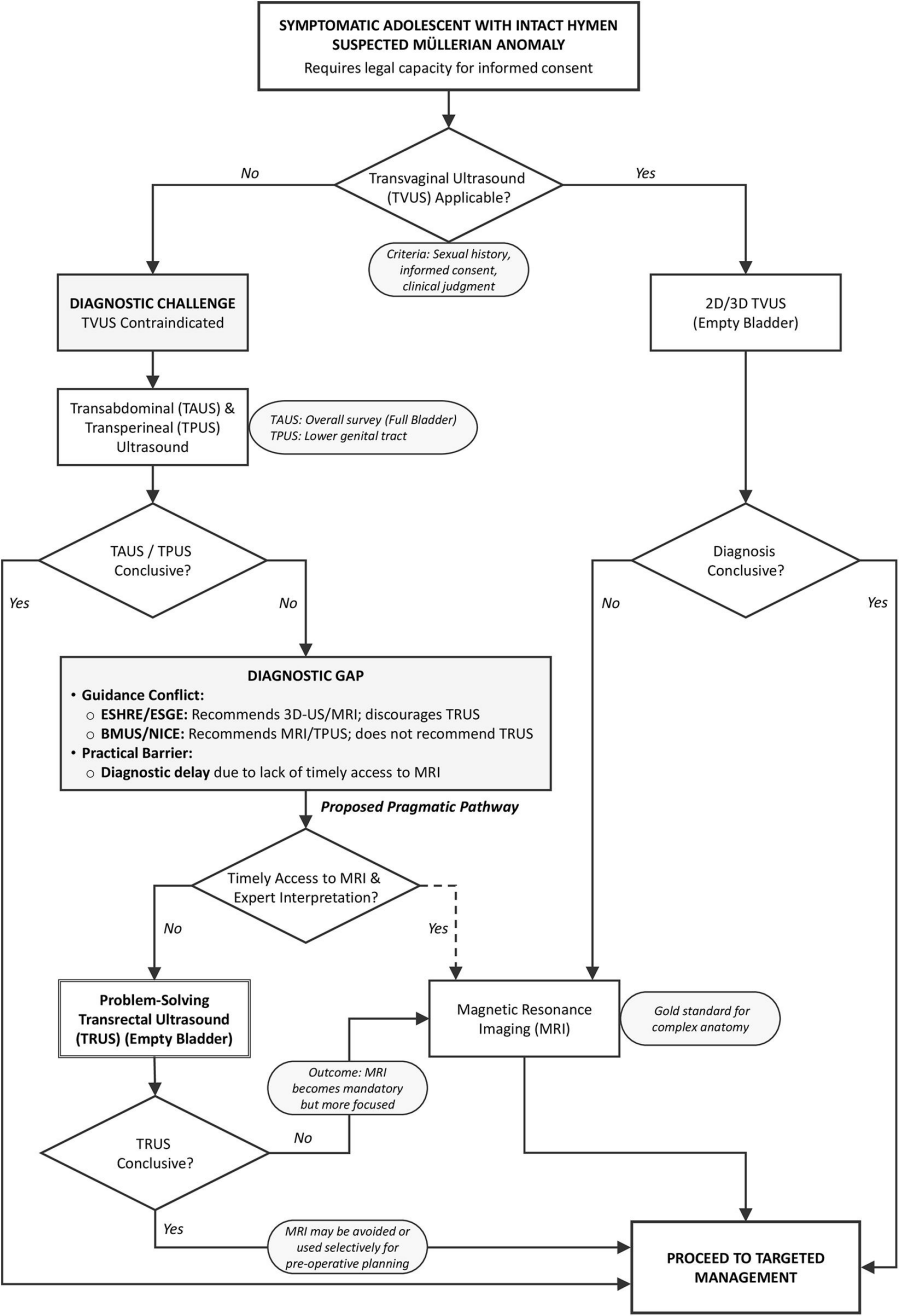
A proposed diagnostic algorithm for suspected Müllerian anomalies in older adolescents. This algorithm presents a pragmatic pathway for adolescents with suspected Müllerian anomalies when transvaginal ultrasound (TVUS) is contraindicated. It addresses the diagnostic gap created by conflicting international guidance and the practical limitations of MRI. The pathway positions transrectal ultrasound (TRUS) as a point-of-care, problem-solving tool to be used after inconclusive non-invasive ultrasound (TAUS/TPUS), aiming to reduce diagnostic delay and refine the need for MRI. Crucially, the pathway to TRUS includes a strict ‘Consent Gate': it is only applicable to older adolescents (e.g., ≥16 or 18 years) with the legal and psychological capacity for informed consent. For younger adolescents (e.g., <16 years without established Gillick competence), the pathway strictly defaults to MRI. Abbreviations: BMUS, British Medical Ultrasound Society; ESHRE, European Society of Human Reproduction and Embryology; ESGE, European Society for Gynaecological Endoscopy; MRI, magnetic resonance imaging; NICE, National Institute for Health and Care Excellence; TAUS, Transabdominal Ultrasound; TPUS, transperineal ultrasound; TRUS, transrectal ultrasound; TVUS, transvaginal ultrasound.

The conflicting recommendations from ESHRE/ESGE and BMUS create uncertainty. To navigate this, we propose a pragmatic, sequential diagnostic algorithm that clarifies the role of each imaging modality (Figure 3). In this proposed pathway, TRUS is not viewed as a routine alternative to TVUS, but as a second-line, problem-solving tool for a specific indication. This indication is strictly defined for the symptomatic older adolescent with an intact hymen who possesses the legal capacity for informed consent, and where: TVUS is contraindicated, initial non-invasive ultrasound (TAUS/TPUS) is non-diagnostic, and MRI is either unavailable, inconclusive, or contraindicated. This positioning respects the principle of using the least invasive method first (TAUS and TPUS) while providing a practical pathway when it fails. In low-resource settings, this pathway may become the immediate next step after an inconclusive TAUS, preventing significant diagnostic delays and allowing for timely intervention, thereby mitigating risks of long-term complications like endometriosis and infertility (7, 21).

### Patient perspective

The patient shared that the uncertainty of her diagnosis had been a major source of anxiety. Regarding the TRUS procedure, she reported that while the idea of a rectal examination was initially daunting, the actual experience was comparable to the discomfort of mild pelvic cramping and was highly manageable. She emphasized that the immediate visualization of the anomaly provided a psychological turning point, validating her pain and building trust in the proposed surgical plan.

## Conclusions

This case, representing a prototypic clinical scenario, does not present a novel technique but rather highlights an unintended consequence of current international guidance for Müllerian anomalies to address real-world diagnostic challenges. When performed with informed consent in appropriately selected patients, TRUS can be a reliable and safe diagnostic option in appropriately selected, consenting older adolescents where transvaginal access is unfeasible and other modalities are non-diagnostic. In healthcare settings where access to MRI is limited, TRUS offers a pragmatic, anatomically appropriate alternative for timely triage and surgical planning. This case supports the consideration of TRUS as a potential problem-solving modality in symptomatic adolescent patients with an intact hymen, particularly in low-resource settings. Furthermore, it underscores the clear need for more consistent and context-sensitive guidance on its role in gynecologic imaging pathways, and serves as a proof-of-concept that strongly justifies further formal research in this population.

## Data Availability

The original contributions presented in the study are included in the article, further inquiries can be directed to the corresponding author.
